# Cor Adiposum as the Cause of Sudden Cardiac Death: A Case Report and Literature Review

**DOI:** 10.7759/cureus.55561

**Published:** 2024-03-05

**Authors:** Emily Dinning, Srinuvasula Muttana, Svetoslav Bardarov

**Affiliations:** 1 Pathology, Richmond University Medical Center, New York, USA; 2 Pathology and Laboratory Medicine, Richmond University Medical Center, New York, USA

**Keywords:** mediastinal lipomatosis, epicardial adipose tissue, cardiac sudden death, anatomical pathology, cardiology research

## Abstract

We present a case of sudden cardiac death in a 65-year-old patient who came to the emergency room with shortness of breath. The gross examination of the heart was significant for extensive left ventricular lipomatosis in association with myocardial fibrosis. Microscopic examination revealed extensive fatty replacement of the myocardial tissue throughout the entire thickness of the ventricular wall (transmural lipomatous myocardial remodeling). We suggest using the term "cor adiposum" to categorize this morphological condition when the transmural lipomatous transformation of the myocardium is present. The fatty replacement of the heart muscle would have led to impaired cardiac function, ultimately resulting in sudden cardiac death in this patient. We also hypothesize that the accumulation of fat in the myocardium might be a compensatory process to preserve ventricular wall compliance.

## Introduction

Cardiovascular diseases remain a leading cause of morbidity and mortality in the United States, killing 375,476 people in 2021 according to the Centers for Disease Control and Prevention, with one in five heart attacks being silent and the person is not aware of it [1]. Atherosclerotic cardiovascular disease commonly underlies acute myocardial infarction (AMI) and sudden cardiac death. While extensive research has explored the pathophysiology of cardiovascular diseases, one overlooked aspect is the role of epicardial fat (EF) and myocardial adipocyte remodeling. This overlooked facet bears significant implications for disease progression.

In this case report, we hypothesize that the transmural fatty infiltration of the myocardial wall may be an adaptive mechanism, resulting from the combined effects of chronic ongoing myocardial ischemia along with bioactive factors released from the overlying EF, promoting myocardial tissue remodeling.

## Case presentation

In this study, we present a case involving a 65-year-old African American male who sought care, for shortness of breath, in the emergency department (ED). While in the ED, the patient expressed a preference for a COVID-19 test and requested discharge due to heightened anxiety associated with the ED environment. The patient reported a history of prior chest pain, although he denied experiencing chest pain on the day of presentation. Laboratory tests, including screening for influenza type A, influenza type B, respiratory syncytial virus (RSV), and SARS-CoV-2, all yielded negative results. Subsequently, the patient declined further evaluation or testing and opted for discharge against medical advice. Shortly after discharge, on the way home, the patient experienced dizziness, lost consciousness, and was pronounced dead upon arrival at the ED.

**Figure 1:**
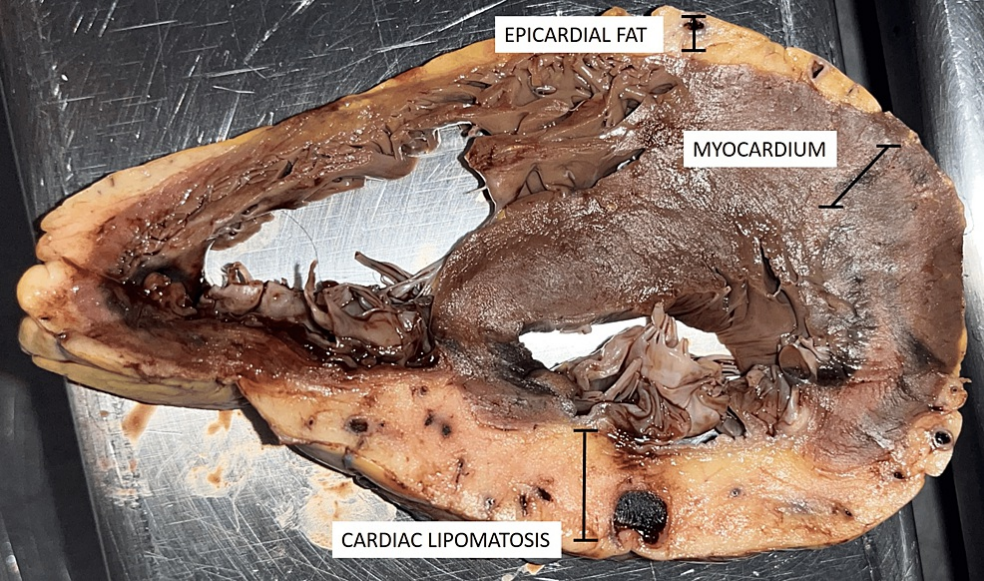
Transmural fat infiltration of the left myocardial wall, arising in a context of extensive myocardial infarction (gross photograph)

Gross examination revealed a heart of normal size and shape, weighing 150 grams (adult male: 90-600 grams). The valve circumferences were as follows: tricuspid valve diameter 5.5 cm (diameter: 3.6-4.6 cm), pulmonary valve 6.6 cm (6.1-7.1 cm), mitral valve 9.5 cm (9.4-9.9 cm), and aortic valve 6.7 cm (6.0-7.4 cm), all without observable calcifications. The right ventricular wall measured 0.5 cm (0.25-0.50 cm), and the left ventricular wall measured 1.8 cm (adult male 65+: 1.0-1.25 cm). The epicardium and pericardium were unremarkable upon gross examination. The pectinate muscles appeared normal, and the coronary ostia were patent. Sections through the myocardium demonstrated extensive fibrosis measuring 4.5 cm, as well as extensive myocardial lipomatosis involving the left ventricular wall, septum, left papillary muscle, and right ventricular wall (Figure 1).

**Figure 2:**
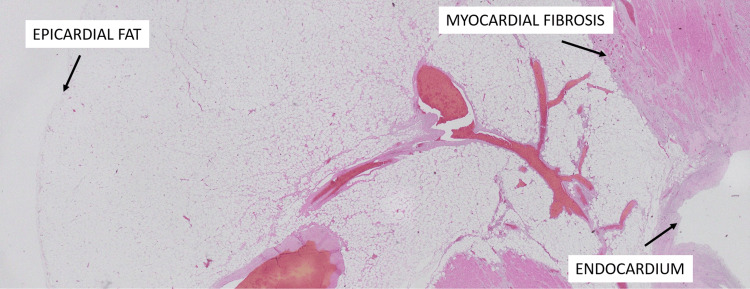
Transmural myocardial adipocytic wall remodeling arising in the context of myocardial ischemia with extensive fibrosis (2x, hematoxylin and eosin (H&E) stain).

Microscopic examination of the left ventricular wall showed extensive transmural myocardial fibrosis and diffuse myocardial lipomatosis extending into the papillary muscles and right ventricular wall. Multiple cross-sections of the coronary arteries revealed focal atheromatous changes without evidence of thrombosis or plaque rupture (Figure 2).

## Discussion

EF is a visceral fat deposit between the visceral pericardium and myocardium that is derived from the same embryological origin of omental and mesenteric adipose tissues with similar physiologic properties to abdominal visceral adipose tissue. Because there is no fascial layer separating the EF from the myocardium, adipocyte infiltration into the myocardial wall and triglyceride infiltration into myocytes is possible. The accumulation of excess cytokines, free fatty acids, and bioactive molecules in cardiac myocytes leads to structural and electrical changes, resulting in remodeling similar to microfibrosis. This remodeling can eventually cause changes in conduction and propagation, leading to lipotoxic cardiomyopathy and arrhythmogenicity. Fatty infiltration of myocytes allows for the creation of specific adipose tissue depots, each with its own secretory metabolic environment. These adipocytic depots could also release bioactive substances, such as adipocytokines, adiponectin, resisting, fatty acid binding protein, leptin, angiogenic factors, remodeling factors, and inflammatory cytokines, all of which are implicated in various heart diseases, including myocardial wall remodeling. The secretory environment created by the individual adipose tissue depots has different effects, depending on the biofactors released and cell types and their responses; thus, the type of cardiac remodeling that occurs is unique to each tissue depot [2].

Multiple imaging modalities can be used to identify and quantify EF, such as echocardiography, which can be supplemented by magnetic resonance imaging (MRI). EF is recognized as a hypoechoic area located in front of the right ventricular wall, and it is difficult to delimit by echocardiography; thus, the thickness is derived by measuring the distances between the epicardium and parietal pericardium. Echocardiography has some limitations due to spatial variations; however, a mean cutoff value to define an increased EF in low-risk populations has been identified to be >5 mm. The current literature concludes that a mean cutoff of >5 mm of EF thickness or a volume of >125 mL is considered abnormal. EF is associated with various heart conditions, especially coronary artery disease and metabolic syndrome, and has many implications clinically that require more research [4].

EF volume appears to be dynamic in its accumulation; there is a selective increase in EF volume in regions of myocardial ischemia while regions of myocardium with normal perfusion or scarring are characterized by a lower EF volume. There is a predisposition for apical areas of the heart to develop increased EF fat volume, which could be due to myocardial ischemia that develops distal to the territories of ischemic vessels. EF content is associated with atherosclerotic coronary artery disease, metabolically active coronary lesions, plaque rupture, and increased clinical events, potentially due to altered metabolic requirements of the myocardium [5].

Our case is similar to the one described by Kanchan et al. [6], and it is that of a patient with extensive transmural myocardial fibrosis and myocardial lipomatosis of the left ventricular wall, left papillary muscle, and right ventricular wall. Focal lipomatous metaplasia at a scar associated with chronic ischemic heart disease is a common transformation, shown to be among 68% of patients [7]. While lipomatosis of the interatrial septum can be a normal finding with aging, the thickness of fat collection >2 cm is associated with atrial arrhythmias and heart block [8].

There are many possible mechanisms as to how EF can lead to increased arrhythmogenicity, including fatty infiltration, fibrosis, and inflammation, which have downstream effects that lead to a pro-arrhythmic environment. The accumulation of EF leads to direct adipocyte infiltration into the myocardium including the atria. The mechanical separation of myocytes by adipocytes can lead to conduction slowing and anisotropy and can promote conduction heterogeneity. Fibrosis is facilitated by the accumulation of metabolically active adipokines secreted by EF. Adipokine activin A, a member of the TGF- family, is secreted by EF and can reproduce atrial fibrosis. Other inflammatory adipokines secreted by EF that lead to atrial fibrosis and are upregulated in atrial fibrillation include matrix metalloproteinases 2 and 7, which regulate extra-cellular matrix activity, and TGF-Beta1. Other pro-inflammatory markers secreted by EF that lead to the remodeling of the myocardium and facilitate arrhythmogenesis include C-reactive protein, IL-6, IL-8, IL-1Beta, and TNF-alpha. These markers are also associated with the incidence, severity, and recurrence of atrial fibrillation.

EF is also a source of reactive oxygen species, which may be a source of arrhythmogenesis. Interestingly, only peri-atrial EF expresses genes involved in oxidative phosphorylation, cell adhesion, cardiac muscle contraction, and calcium signaling pathways, as opposed to peri-ventricular and peri-coronary EF, suggesting that atrial fibrillation can develop due to specific characteristics of depots of EF. In addition to other mechanisms, autonomic dysfunction contributes to the development of arrhythmias. There are ganglionated plexi within the EF, which can cause dysfunction of the parasympathetic and sympathetic nervous systems and result in shortened action potential duration and increases in calcium transient in the atrial myocardium, initiating and perpetuating atrial fibrillation. EF has also been associated with diastolic dysfunction due to previously mentioned mechanisms or due to physical compression of the left ventricle in particular. A final method in which EF could contribute to arrhythmogenicity is due to its correlation with high dominant electrical frequency, suggesting that it is associated with triggers, which are localized sources of spontaneous, rapid, and repetitive electrical activities, especially from the pulmonary vein that can initiate and maintain atrial fibrillation [9].

In this case report, we hypothesize that extensive chronic ongoing myocardial ischemia can result in extensive scarring and reduced compliance of the myocardium. Thus, the transmural infiltration of adipose tissue may represent a compensatory mechanism to help preserve wall compliance. It seems that the EF might be directly involved in this tissue remodeling, but more research is needed to establish this as a fact.

## Conclusions

Considerable confusion has arisen from varied terminologies used to describe fatty changes in the heart, such as adipocytic metaplasia, fatty degeneration, or lipoid dysplasia. We propose using the term "cor adiposum" when echocardiography or radiologic studies show transmural replacement of the left myocardium by adipose tissue with corresponding ECG changes, or when this phenomenon is observed post-mortem in cases of sudden arrhythmogenic death. The term "cardiac lipomatosis" should refer to increased EF without demonstrable transmural myocardial infiltration. Standardized terminology will improve consistency in reporting and allow better comparison of outcomes across studies of fatty cardiac changes. However, more research is needed to determine if echocardiographic measures of adipose infiltration or replacement of myocardium can help predict arrhythmogenicity and sudden cardiac death risk.
